# Fatigue Life Prediction for Injection-Molded Carbon Fiber-Reinforced Polyamide-6 Considering Anisotropy and Temperature Effects

**DOI:** 10.3390/ma17020315

**Published:** 2024-01-08

**Authors:** Joeun Choi, Yohanes Oscar Andrian, Hyungtak Lee, Hyungyil Lee, Naksoo Kim

**Affiliations:** 1 Department of Mechanical Engineering, Sogang University, Seoul 04107, Republic of Korea; 2 Polymer R&D Team, GS Caltex R&D Center, Daejeon 34122, Republic of Korea

**Keywords:** fiber-reinforced composite, injection molding process, numerical simulation, anisotropy, temperature effect, fatigue life prediction

## Abstract

The effects of anisotropy and temperature of short carbon fiber-reinforced polyamide-6 (CF-PA6) by the injection molding process were investigated to obtain the static and fatigue characteristics. Static and fatigue tests were conducted with uniaxial tensile and three-point bending specimens with various fiber orientations at temperatures of 40, 60, and 100 °C. The anisotropy caused by the fiber orientations along a polymer flow was calculated using three software connecting analysis sequences. The characteristics of tensile strength and fatigue life can be changed by temperature and anisotropy variations. A semi-empirical strain–stress fatigue life prediction model was proposed, considering cyclic and thermodynamic properties based on the Arrhenius equation. The developed model had a good agreement with an *R*^2^ = 0.9457 correlation coefficient. The present fatigue life prediction of CF-PA6 can be adopted when designers make suitable decisions considering the effects of temperature and anisotropy.

## 1. Introduction

Fiber-reinforced plastics (FRPs) are widely utilized as structural materials in the aerospace and automotive industries due to their low density and high specific strength [1,2,3,4,5,6]. The injection molding process efficiently produces FRP material, facilitating the manufacturing of complex geometries and ultimately increasing the material production rate [7]. The manufacturing conditions and operating environments influence the failure behavior of injection-molded FRPs. The complex fiber orientation distribution, determined by the polymer flow path at different locations, substantiates the considerable anisotropic behavior of FRPs [8]. In addition, the tensile strength strongly differs at different temperatures. Consequently, the need to characterize the combined effects of anisotropy and temperature is a pressing theme in assessing the static and fatigue behavior of FRPs [9].

The influence of fiber orientation distribution, fiber length, and fiber volume fraction on mechanical strength and stiffness, mechanical elasticity, and anisotropy has been extensively investigated [10,11,12,13,14,15,16,17,18,19]. Brighenti et al. [13] evaluated the static and fatigue behavior of short fiber-reinforced plastics utilizing a micromechanical model based on a Gaussian-like distribution function using the average fiber orientation. Just et al. [14] developed a crack growth model depending on the degree of anisotropy and fiber orientations, predicting the crack growth path with good agreement.

Most of the fracture analysis for FRP has been proposed at room temperature, and fracture behavior considering the effect of various temperatures has been studied far less. The bonding ability at the interface of FRP is weakened by increased temperature. As a result, the debonding length of the fibers is increased, and the tensile strength is partially reduced [20,21,22,23,24,25]. Li et al. [26] proposed a theoretical model to predict the longitudinal tensile strength of FRP under various temperatures. Kawai et al. [27] investigated the temperature dependence of static strength employing Arrhenius-type equations and clarified that the decrease in compressive strength with increasing temperature is smaller than that in tensile strength.

As concerns the fatigue life prediction models, theory-based formula models such as the Manson–Coffin model [28] and the SWT model [29] have been used for various composite materials, modified to consider the effects of anisotropy [30,31,32,33,34,35] and temperature [36,37,38,39,40]. Regarding FRP, Launay et al. [34] developed a predictive fatigue criterion based on the dissipated energy density per cycle of polyamide 66 base polymer filled with glass fibers, contemplating both anisotropic and nonlinear behavior. Fouchier [41] proposed an energetic fatigue criterion of injection molded short fiber-reinforced plastics at 100 °C.

Although the fracture behavior and fatigue life have been studied, none seem to deal with the fatigue fracture analysis combining the effects of anisotropy and temperature of injection-molded short fiber-reinforced plastics. The application of FRP, which combines temperature conditions with fiber orientation distribution, means that the influence of environmental temperature and random locations of load impact regions can be considered.

For these reasons, we investigated the static and cyclic fracture behavior of 20% volume fraction CF-PA6 material at three different temperatures along 0° (injection direction), 45°, and 90° direction. All specimens were machined on injection-molded plates to analyze the anisotropic behavior caused by fiber orientation distribution through polymer flow. Short fiber-reinforced plastic manufactured by the injection process has an arbitrary fiber orientation in the center of thickness and a fiber arrangement parallel to the polymer flow direction when it deviates from the center [42,43]. The direction of the specimen having a fiber orientation arranged parallel to the polymer flow direction was designated as 0° to evaluate the difference in mechanical properties due to the relationship between fiber orientation and principal stress, and the directions of 45° and 90° were determined in consideration of the angle during specimen processing.

The three experimental temperatures (40, 60, and 100 °C) adopted in static and fatigue tests were determined in consideration of the fact that the operating environment temperature of CFRP applied to various products such as automotive, aircraft, and solar panels exceeds room temperature and the glass transition temperature of the material, 64 °C [44].

Three commercial software (Moldflow Insight 2019, Autodesk Helius 2019, and Abaqus 2020) have been utilized to describe the fiber orientation distribution from a numerical point of view, showing a maximum difference between finite element analysis and experimental load–stroke curve integrals equal to 5.12%.

To enhance the mechanical reliability of CF-PA6, a semi-empirical fatigue life prediction model based on the strain–stress-based fatigue failure theory has been developed and verified using tensile and three-point bending test specimens. The proposed model can predict the failure cycle with high accuracy and ensures the reliability of injection-molded short fiber-reinforced plastic products under high temperatures and various load conditions. The developed semi-empirical fatigue life prediction model shows a high correlation coefficient of *R*^2^ = 0.9457. The results provide the possibility of predicting the fracture behavior considering the anisotropic behavior and temperature effect of CF-PA6 with the proposed model.

## 2. Fatigue Life Prediction Procedure

**Figure 1:**
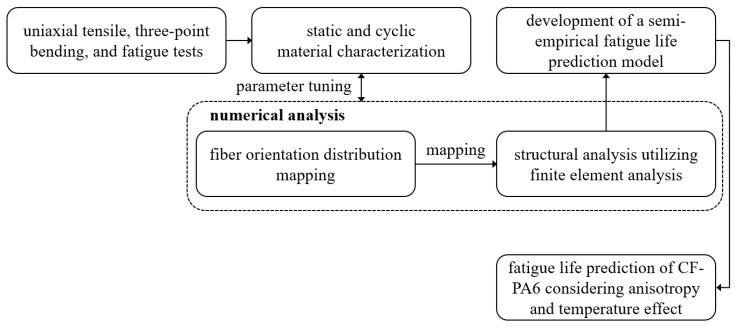
Fatigue life prediction procedure of CF-PA6 based on the semi-empirical model.

Fatigue life prediction considering the anisotropy and temperature effect of 20% short carbon fiber volume fraction CF-PA6 material has been conducted through the procedure shown in Figure 1. The fiber volume fraction is calculated based on the Rule of Mixtures. The uniaxial and three-point bending static and cyclic tests were carried out to evaluate the mechanical properties. The specimens were cut from the injection-molded plate in three directions, and experiments were conducted at three different temperatures.

The test results obtained through the machined specimens and selected test conditions were used to characterize the mechanical properties of CF-PA6. In addition, the obtained load–displacement curve was adopted to adjust the material parameters used for numerical analysis, and the stress–strain curve was analyzed to confirm brittle properties and yield strength. Finally, cycle behavior was evaluated from fatigue tests under various load-controlled test conditions.

The numerical analysis models were reverse-engineered through static experiments to apply the semi-empirical model’s stress, strain, and temperature values. The model was divided into two categories: a model for calculating fiber orientation distribution and a model for structural analysis. The entire numerical analysis procedure is constructed through three commercial software.

The semi-empirical model based on the life prediction model utilizing strain amplitude was developed and modified by formalizing an effect depending on temperatures and directions. The finite element method extracted the minimum and maximum stress and strain values in the stabilized hysteresis loop. The given values were used to input the proposed semi-empirical model and the fracture strain and stress in the uniaxial tensile experiments.

## 3. Material Characterization

### 3.1. Static Mechanical Properties Characterization

**Figure 2:**
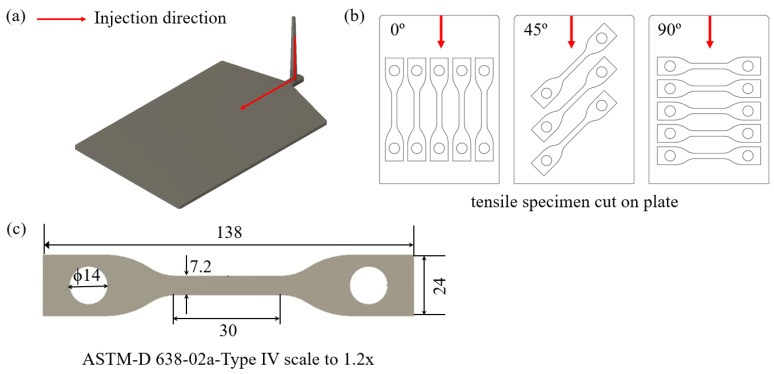
(**a**) The injection-molded plate with a runner. (**b**) 0°, 45°, and 90° directions-cut ASTM-D 638 specimens. (**c**) The dimensions of 1.2 times ASTM-D 638 specimen.

The pellets used to manufacture tensile and three-point bending specimens for mechanical characteristics of 20% volume fraction short CF-PA6 were produced utilizing a mixture of PA6 and mono-carbon fibers. From the four mm-thick injection-molded plates, the tensile test specimens were cut along 0 (injection flow direction), 45°, and 90° directions according to the ASTM-D 638-02a-TYPE IV specification, as shown in Figure 2. The ASTM-D 638 specimens were manufactured 1.2 times larger than the standard in the 14 mm diameter hole made for the fixture on the heat chamber jig. Therefore, the fracture could be well-achieved in the center of the specimen through dimensional adjustment. The gauge length of the 1.2 times-larger ASTM-D 638 specimen was 30 mm.

**Figure 3:**
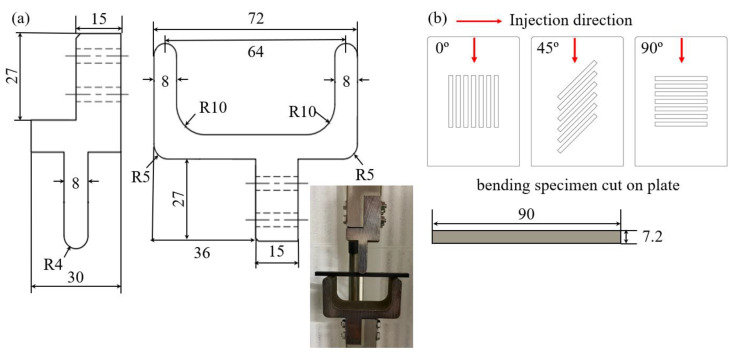
(**a**) Test jig dimensions for the upper part (**left**), lower part (**right**), and experimental setting for three-point bending test in an environmental chamber. (**b**) Schematic dimensions of injection plates for extracting three-point bending test specimens and specimen shape and specifications with ASTM-D 790.

For checking the stress triaxiality on the material, the three-point bending test jig was designed to match the dimension of the room inside the heating chamber, which was 300 mm in height and 100 mm in diameter. In addition, the static and cyclic behavior under complex stress conditions were analyzed by machined three-point bending specimens, similarly cut in three directions, to investigate the anisotropy caused by fiber orientation distribution, as shown in Figure 3.

The temperature expansion inside the heating chamber was investigated in the preliminary stage. Thermocouples were attached to the jig and the specimen to observe the thermal expansion by recording the temperature by heating time. As a result, target temperatures of 40, 60, and 100 °C were maintained stable with a range of ±5 °C after 30, 20, and 15 min, respectively. By temperature tracking, all experiences were carried out at stable temperatures by comparing temperature recordings between repeated tests.

Tensile experiments and three-point bending tests were conducted at a tensile and bending speed of 2 mm/min. All tests were performed with the heating chamber maintained at a constant operating temperature of 40, 60, and 100 °C. The specimens were dried at 100 °C for 1 h with 12 m^3^/h heat flow in an oven dryer to remove the humidity before proceeding with experiments. All static experiments were repeated three times to increase experimental accuracy. The strain was obtained by means of a 20 mm gauge length extensometer in the case of the uniaxial tensile test.

### 3.2. Cyclic Mechanical Properties Characterization

Fatigue experiments were conducted based on load control with the same temperature conditions and specimens as the tensile and three-point bending tests. The mean loads of fatigue tests are summarized in Table 1, considering the total stress range. The same mean load was used for three maximum and minimum load combinations.

In addition, complete elasticity and complete plastic deformation region conditions were utilized. All fatigue tests were carried out with a frequency of 1 Hz. In addition, three-point bending fatigue experiments were conducted to consider complex stress states. Fifty-four fatigue test data of uniaxial tensile and three-point bending specimens were obtained for five and three conditions considering each temperature and specimen direction. For the reliability of the experimental results, all three repetitive tests were performed for the fatigue test of each load case, and the intermediate value was adopted and used as the result. The mean and amplitude loads were selected to consider both low-cycle and high-cycle fatigue failure. In addition, the cycle when the load decreases to 40% or less of the previous cycle due to damage to the specimen was selected as the failure cycle. The results of each condition are summarized in Table 2 and Table 3.

**Figure 4:**
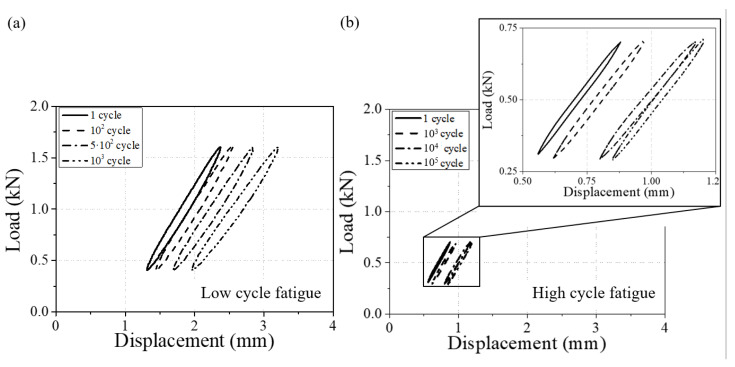
Test results of hysteresis loops for low- and high-cycle fatigues (90° and T = 60 °C). (**a**) fatigue failure at 3800 cycles (0.6 kN load amplitude and 1.0 mean load) and (**b**) fatigue failure at 346,900 cycles (0.2 kN load amplitude and 0.5 mean load).

In the initial stage of the fatigue test, the hysteresis loop shows instabilities due to experimental settings. For this reason, the stress and strain were calculated in the last stabilized cycle, where the maximum and minimum displacements differ by less than 5% from the fatigue fracture cycle’s hysteresis loop. The periodic spectrum of fatigue life was considered from 10^2^ to 10^6^. The hysteresis loop moves in a positive direction of strain as the load amplitude increases, indicating that the degree of asymmetry intensifies. An example of a hysteresis loop of one low-cycle fatigue and one high-cycle fatigue is reported in Figure 4.

### 3.3. Numerical Analysis

A numerical analysis model was constructed to utilize the fatigue test results in the semi-empirical model and to investigate the cyclic mechanical properties of CF-PA6 under complex stress conditions. The three-point bending test to examine fracture behavior in complex stress states obtained true stress and strain through numerical analysis. Three commercial software were used to account for the influence of the fiber orientation. The injection molding process was simulated using Autodesk Moldflow Insight/Synergy (AMI), and the fiber orientation distribution was calculated at the end of the cooling phase.

**Figure 5:**
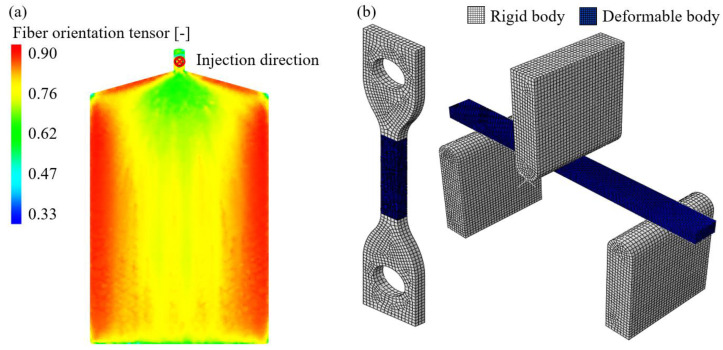
(**a**) Fiber orientation distribution from AME. (**b**) Computational domain for structural analysis for the uniaxial specimen (**left**) and the three-point bending specimen (**right**).

The accuracy of the fiber orientation distribution from Moldflow was evaluated by comparing the fiber alignment from the 3D X-ray CT (XCT) results, as shown in Choi et al. [33]. The obtained optimal RSC parameter utilizing the XCT data in a previous study [33] was adapted to the Moldflow simulation to calculate the fiber orientation distribution accurately. The XCT data were taken with 140 kV voltage and 2 μm pixel size. One injection gate was used for the injection molding simulation. The injection and cooling times were 2 and 20 s for the analysis, and the mold and melt temperatures were 85 and 285 °C in the simulation. Each element’s mechanical properties were mapped to the structural simulation mesh using Advanced Material Exchange Helius 2019 (AME) in the second step. Finally, the Abaqus input files with the inbuilt element-based mesh sets were created. This procedure is essential since the fiber orientation results affect the static and cyclic mechanical properties. The developed numerical analysis models for fiber orientation distribution calculation with AMI, AME, and structural analysis with Abaqus are shown in Figure 5a and b, respectively.

The fiber orientation tensor was calculated using the Folgar–Tucker orientation model in Moldflow simulation, as shown in Equation (1). $a_{ij}$ is the fiber orientation tensor, $0.5\omega_{ij}$ is the vorticity tensor, $0.5{\dot{\lambda }}_{ij}$ is the deformation rate tensor, and *C_I_* is the fiber interaction coefficient.

(1) $$\frac{Da_{ij}}{Dt}=-\frac{1}{2}\left(\omega_{ik}a_{kj}-a_{ik}\omega_{kj}\right)+\frac{1}{2}\lambda \left({\dot{\gamma }}_{ik}a_{kj}+a_{ik}{\dot{\gamma }}_{kj}-2a_{ijkl}{\dot{\gamma }}_{kl}\right)+2C_{l}\dot{\gamma }\left(\delta_{ij}-3a_{ij}\right)$$

The Ramberg–Osgood flow stress model was combined with a modified Hill′ 48 yield function to account for the influence of the fiber orientation on the mechanical properties of the CF-PA6. The equations of the Ramberg–Osgood flow stress model and a modified Hill′ 48 yield function are shown in Equation (2). The definition of model parameters and the optimized Ramberg–Osgood model constants for the CF-PA6 are summarized in Table 4 and Table 5.

(2) $$\begin{array}{l} \sigma =Ε{\;}^{1/n}{\left(Κ\right)}^{\left(n-1\right)/n}{\left(\varepsilon_{p,eff}\right)}^{1/n}\\ \sigma_{eff}=\sqrt{\frac{{\left(\alpha \sigma_{11}-\beta \sigma_{22}\right)}^{2}+{\left(\beta \sigma_{22}-\beta \sigma_{33}\right)}^{2}+{\left(\beta \sigma_{33}-\alpha \sigma_{11}\right)}^{2}+6\left[{\left(\sigma_{12}\right)}^{2}+{\left(\sigma_{23}\right)}^{2}+{\left(\sigma_{31}\right)}^{2}\right]}{2}}\;\\ \alpha \left(\lambda_{I}\right)=\theta +\left[\frac{\left(\alpha_{m}-\theta \right)}{\left(\lambda_{m,I}-1/2\right)}\right]\left(\lambda_{I}-1/2\right),\beta \left(\lambda_{I}\right)=\theta +\left[\frac{\left(\beta_{m}-\theta \right)}{\left(\lambda_{m,I}-1/2\right)}\right]\left(\lambda_{I}-1/2\right) \end{array}$$

The mechanical properties according to fiber orientation were calculated considering anisotropy through the Ramberg–Osgood model on the Moldflow simulation. Each mechanical property was derived through seven constants ranging from σ_0_ to λ_m,I_ in Table 5. *σ*_0_ is the stress level at which plastic strain becomes dominant. Using the experimental results by specimen direction and temperature, the constant with the highest agreement between the analysis and experimental result was derived through reverse engineering. Each constant was determined through the BFGS optimization technique to minimize the area difference between the load–displacement curve FEA result and the experimental result according to each coefficient combination. The orientation and elastic modulus of the fiber had a more significant influence on the calculation of the anisotropic behavior of the material, so the polymer matrix elastic modulus was derived relatively low.

In general, the elastic modulus of carbon fiber is higher than the value obtained through the optimization technique presented in Table 5. However, the Ramberg–Osgood model constants obtained in this study were not calculated by evaluating the elastic modulus of the matrix and the fiber through the experiment. They were derived by reverse engineering so that the difference between the experimental and numerical analysis load was the least in consideration of the influence of each constant on each other in one combination consisting of seven coefficients from *σ*_0_ to *λ*_m,I_. Therefore, the elastic modulus of fiber was obtained lower than the actual fiber value. Even though the elastic modulus was not directly evaluated and used, the coefficient obtained is meaningful because the stress–strain value utilized in predicting fatigue life can be derived through numerical analysis by minimizing the error between analysis and experiment. When an optimization technique is applied by experimentally deriving the elastic modulus of each matrix and the fiber, the constants other than the elastic modulus would have different values in Table 5, and a new combination constant would be derived.

In this study, the accuracy of numerical analysis was considered a significant factor in determining the input variables of the fatigue life prediction model because it is essential to accurately calculate the stress and strain field when subjecting the load. To match the experimental data with numerical analysis results, the reduced strain closer model parameter, ARD-RSC, was optimized with the BFGS optimization module in the Python program. The short fiber flow, which changes fiber orientation distribution, was controlled by tuning the ARD-RSC parameter, allowing proper consideration of complex stress states.

## 4. Fatigue Life Prediction Model

The fatigue life prediction model of CF-PA6 was developed to consider the effects of temperature and anisotropic behavior due to fiber orientation distribution, starting with the strain-based Manson–Coffin model (Equation (3)) [28]. The $\varepsilon_{p,\;\max}$ and $\varepsilon_{p,\;\min}$ are the maximum and minimum plastic strain values. The model correlates the plastic strain amplitude, $\Delta \varepsilon_{p}$, with the failure cycle, *N_f_*, to predict fatigue life through the material coefficients *A* and *c*.

(3) $$f(\varepsilon )\;=\frac{\left(\varepsilon_{p,\;\max}-\varepsilon_{p,\;\min}\right)}{2}=\Delta \varepsilon_{p}=A{\left(N_{f}\right)}^{c}$$

Efforts were made by Choi et al. [31] to express the anisotropic behavior of a directional material by adding a maximum von Mises stress ratio between the angle and 0° directions considered as in Equation (4). $\varepsilon_{f}$ is the fracture strain of tensile experiment for each specimen angle. $\varepsilon_{\max}$ and $\varepsilon_{\min}$ are the total maximum and minimum strain of fatigue test conditions. $\sigma_{peak,\;\;\theta }$ is the maximum von Mises stress and the stress term’s denominator is the value of 0° directions. For the stress term, the anisotropic effect was considered by setting the sine value added by one as an index.

(4) $$f(\varepsilon ,\theta )\;=\;\left(\frac{\varepsilon_{\max}-\varepsilon_{\min}}{2\varepsilon_{f}}\right){\left(\frac{\sigma_{peak,\;\;\theta }}{\sigma_{peak,\;\;\theta =0}}\right)}^{\;(1+\sin\theta )}=A{\left(N_{f}\right)}^{c}$$

In the case of CF-PA6, however, different fiber orientation distributions are shown despite minor position changes due to the injection molding process. If anisotropic behaviors are identified through a ratio between specific and reference directions, predicting critical fracture parts or fatigue life can be significantly reduced. Since the anisotropic behavior of CF-PA6 appears from the relationship between the fiber direction and the principal stress direction, the fatigue life is affected by fiber orientation distribution. Due to the difficulties of considering the fiber orientation distribution in the fatigue life prediction model, numerical analysis or SEM microscopic image photography must be accompanied to calculate the stress and strain. Therefore, the fatigue life prediction model was developed considering the relationship between principal stress and fiber orientation vector in terms of stress rather than the fiber orientation distribution. The model is presented in Equation (5).

(5) $$f(\varepsilon ,\sigma )\;=\;\left(\frac{\varepsilon_{\max,i}-\varepsilon_{\min,i}}{2\varepsilon_{f,\;i}}\right){\left(\frac{\sigma_{\max,i}-\sigma_{\min,i}}{2\sigma_{f,\;i}}\right)}^{\ln\left(\frac{\sigma_{f,\;i}}{\sigma_{\max,\;i}}\right)}\left(e^{-T_{ref}/T_{ope}}\right)=A{\left(N_{f}\right)}^{c}$$

The first term is that of the Manson–Coffin model, and the second term is anisotropic. $\varepsilon_{f,\;i}$ and $\sigma_{f,\;i}$ are the fracture strain and stress of tensile experiments for each specimen direction and temperature condition. $\varepsilon_{\max,\;i}$, $\varepsilon_{\min,\;i}$, $\sigma_{\max,\;i}$, and $\sigma_{\min,\;i}$ are the maximum and minimum total strain and von Mises stress. The anisotropic term includes the influence of FOD through stress values. The effect of anisotropy on fracture failure can be considered effectively in the developed model by combining fracture stress, $\sigma_{f,\;i}$, and maximum stress, $\sigma_{\max,\;i}$, using a logarithmic function. The last term concerns the temperature effect with the reference temperature (*T_ref_*) of CF-PA6, 20 °C, and the experimental temperature in Arrhenius law.

## 5. Results

**Figure 6:**
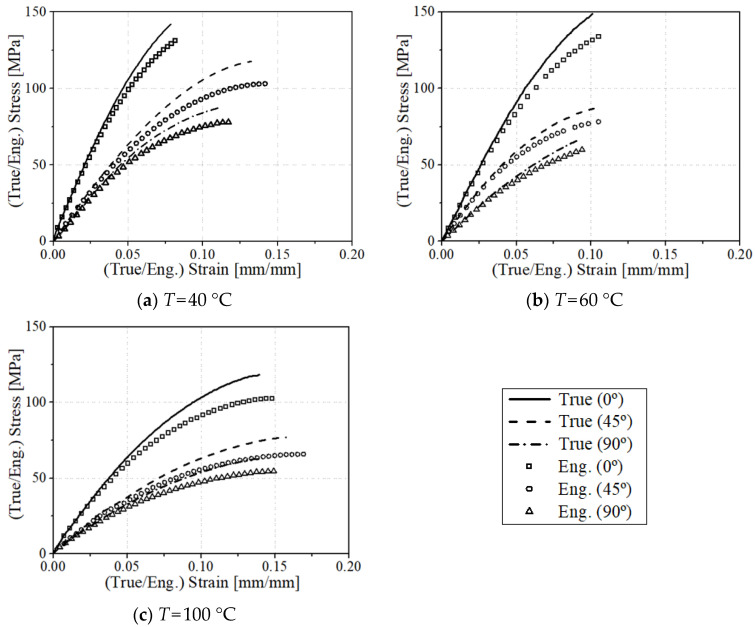
Experimental results of true and engineering (eng.) stress–strain curves for 0°, 45°, and 90° specimens in uniaxial tensile tests.

The true and engineering stress–strain curves of the uniaxial tensile experiment are summarized in Figure 6. The static behavior of CF-PA6 shows a linear elastic stress–strain relationship until fracture. The tensile strengths of CF-PA6 from the uniaxial tensile test are summarized in Table 6 for each direction and temperature. As shown in the load–stroke curve of the uniaxial tensile test, the higher mechanical properties appear when the main load direction and the fiber orientation tensor coincide.

The engineering strain is the changed displacement at each measurement moment divided by the initial gauge length, and the true strain is the changed displacement divided by the displacement immediately before. The engineering strain does not consider the changed length of the specimen, and the true strain takes into account the changed length of the specimen at every moment. The nominal stress is based on the cross-sectional area of the initial specimen when calculating the stress, and the true stress is based on the actual cross-sectional area that continues to change during the tensile test. Just before the specimen is broken, the cross-sectional area becomes very small, and the engineering stress does not consider the decrease in the cross-sectional area, so the closer to the fracture stress, the greater the true stress than the engineering stress, as shown in Figure 6.

In order to minimize the phenomenon that mechanical properties differ due to fiber orientation distribution depending on the location of the injection molded plate from which the specimen was machined, the stress–strain curves were compared by adopting the results of the specimen collected from the center to confirm the tendency by angle and temperature. The average difference in tensile strength due to the specimen location was measured as 10.4%, 8.2%, and 9.7% at 0°, 45°, and 90°, respectively. In order to consider the difference in mechanical properties, structural analysis was performed by mapping the fiber orientation at the location where each sample was machined.

**Figure 7:**
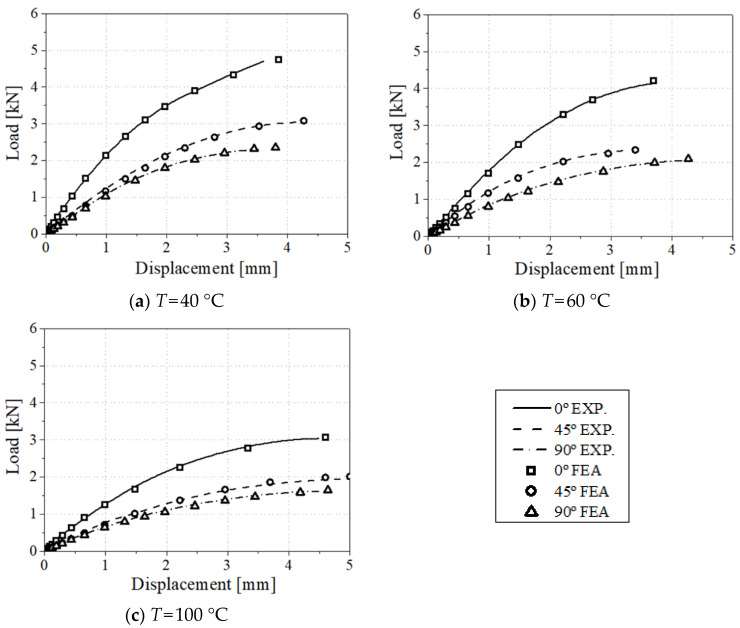
Load–displacement comparison between experimental and FEA results for 0°, 45°, and 90° specimens in uniaxial tensile test conditions.

**Figure 8:**
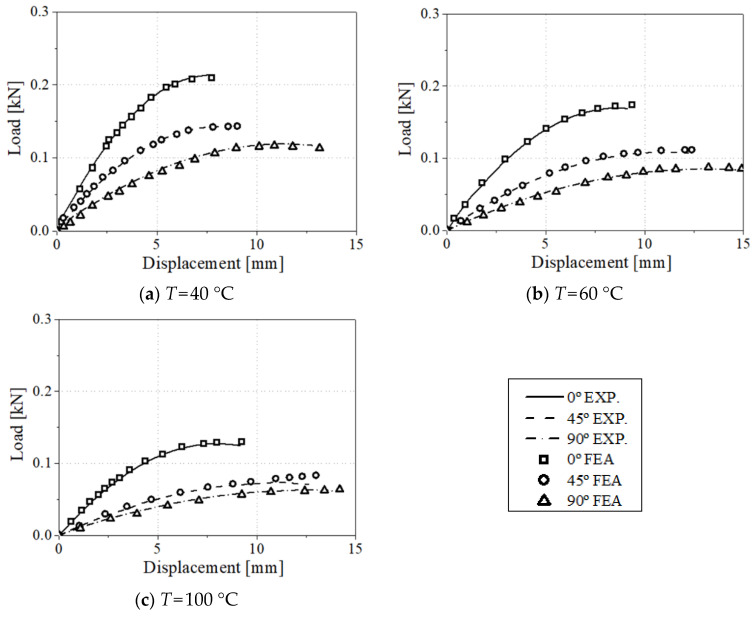
Load–displacement comparison between experimental and FEA results for 0°, 45°, and 90° specimens in three-point bending test conditions.

The load–displacement curve comparison between experimental and FEA results on the uniaxial tensile specimen and three-point bending test is reported in Figure 7 and Figure 8, respectively. The tensile displacements of all the experiments were obtained using the 20 mm extensometer to compare the experiment results and numerical analysis accurately. The numerical analysis results indicate that higher anisotropy is observed according to the test direction with the increase in test temperature. In addition, as the load decreases, the influence of the matrix increases. It can also be confirmed that the elasticity increases as the test direction coincides with the injection direction. For the three-point bending tests, the stress and strain values are calculated by optimizing the material parameters by utilizing the tensile and three-point bending test results together. Implemented numerical models and proposed structural equations were validated and used to explain the anisotropy and temperature effects of CF-PA6.

**Figure 9:**
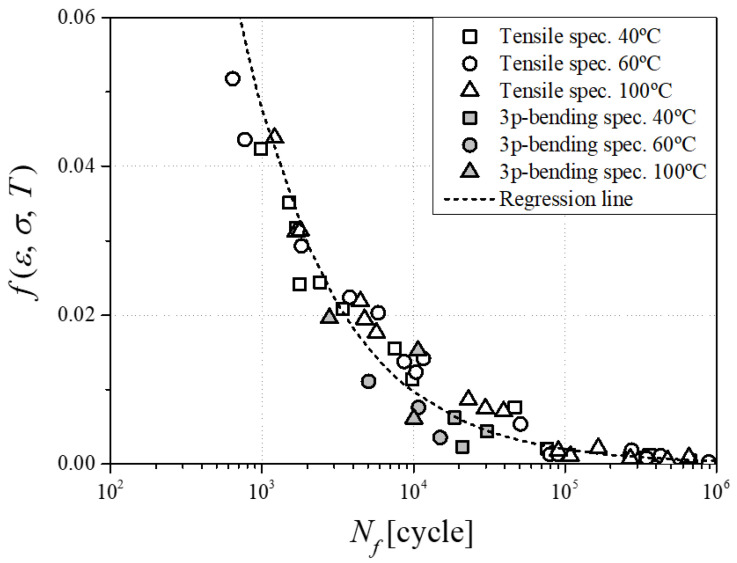
Discrete energy function values from various fatigue tests and their regression line.

The correlation coefficient (*R*^2^) is slightly lower when considering various temperatures simultaneously compared to a single temperature; however, the proposed semi-empirical model confirms that fatigue life expectancy is well predicted. Based on a validated numerical analysis model, as presented in the previous section of the paper, each combination of investigated temperature and specimen direction, the von Mises stress, and strain at the minimum and maximum load associated with the fatigue experiment were exported from the simulation. The temperature was selected as the reference, room, and experimental temperature inside the heating chamber. The constants of the developed model Equation (5) were calculated by *A* = 5.6734 and *c* = −0.692 based on a total set of 54 data, as shown in Figure 9, and the correlation coefficient is *R*^2^ = 0.9457. Thus, the reliability of the proposed function of the anisotropic fatigue test data integration obtained under various stress states and temperatures is demonstrated.

**Figure 10:**
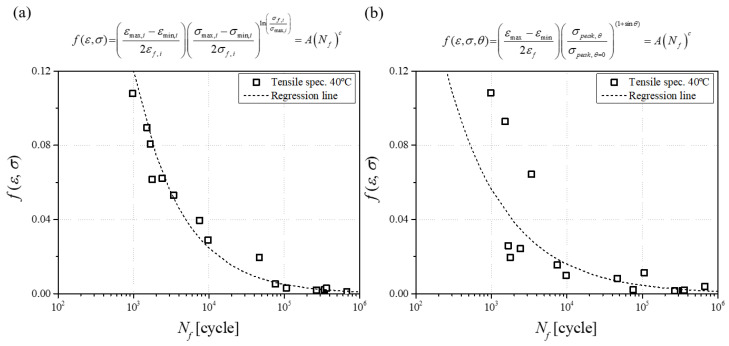
Fatigue experiments regression on (**a**) modified Equation (5) model and (**b**) Equation (4) model.

In order to evaluate the performance of the developed fatigue life prediction model, the regression results of Equations (4) and (5) were compared. From a macroscopic point of view, the tensile fracture strength of 0° was adopted as the denominator of the stress term of Equation (4), and the maximum stress value of each fatigue test was used in the numerator. In addition, since Equation (4) does not consider the temperature effect, Equation (5) was calculated without the last term to compare the model performance in the same environment. As a result of the calculation, it was confirmed that *A* and *c* of Equation (4) and modified Equation (5) were *A* = 2.4959, *c* = −0.549, and *A* = 14.138, *c* = −0.69, respectively. The correlation coefficients were *R*^2^ = 0.9729 and *R*^2^ = 0.6548, respectively, which was further excellent in the performance of Equation (5). It was confirmed that the fatigue life prediction model developed using the principal stress worked well without considering the fiber orientation distribution, which is difficult to calculate, as an angle. The comparison of calculated results is shown in Figure 10.

**Figure 11:**
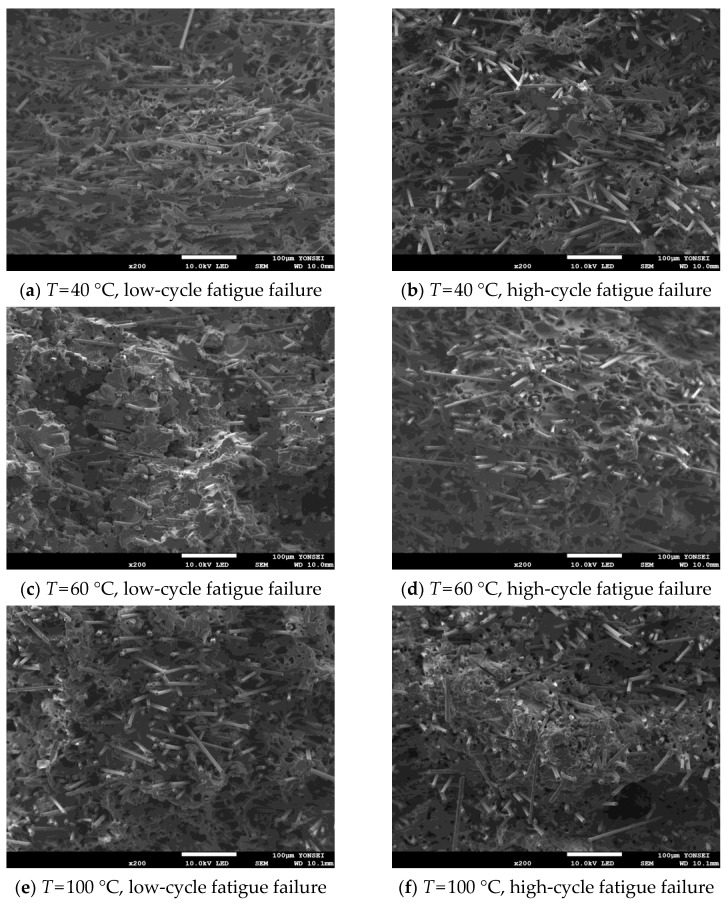
Matrix and fiber at the failure surface in case of low- and high-cycle fatigue.

**Figure 12:**
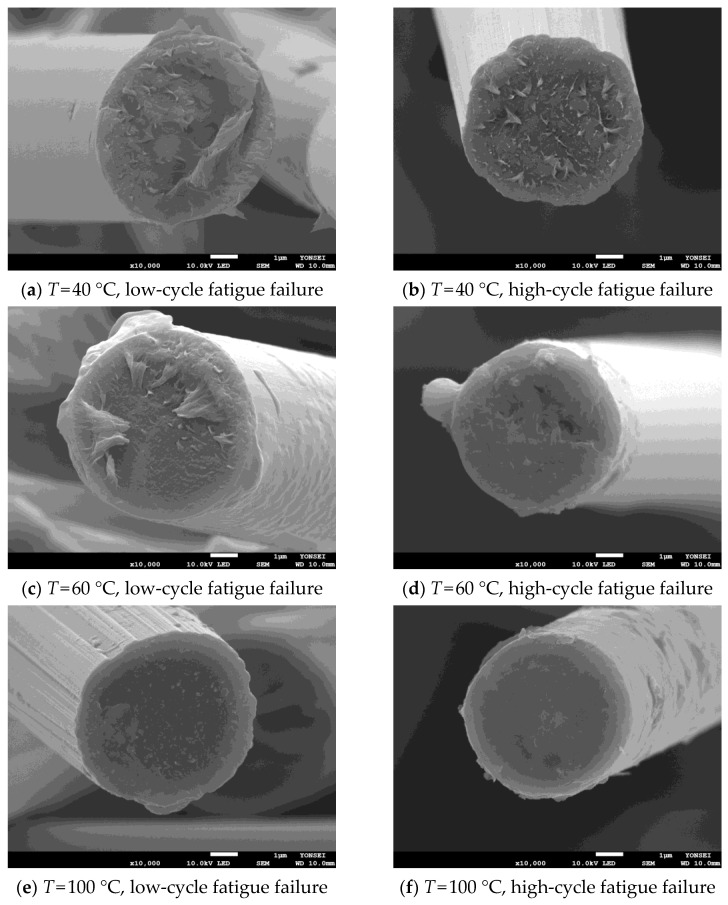
Detail of fiber’s failure surface in low- and high-cycle fatigue.

Moreover, the fracture behavior of the matrix and fiber according to low- and high-cycle fatigue fracture by test temperature was analyzed through SEM analysis. The SEM image was obtained at a magnification at which the matrix and the fiber could be observed simultaneously, and the result of enlarging a single fiber for the analysis of the fracture surface of the fiber was also taken. Each result is reported in Figure 11 and Figure 12.

As shown in Figure 11, high-cycle fatigue shows more significant irregularities in the base material compared to low-cycle fatigue. From these results, it can be confirmed that the polymer matrix causes more significant deformation as the fatigue failure cycle number increases. This result is related to the fact that the shorter the fatigue fracture cycle, the more the load is transmitted by the fiber. In addition, it can be seen that the fiber remains longer as the temperature increases, and the matrix is broken. Figure 12 shows that the higher the test temperature and the longer the fracture cycle, the more even the fracture surface of the fiber is.

## 6. Conclusions

- This study predicted the fatigue life expectancy of CF-PA6, a plastic reinforced with short fiber, through a strain-based semi-empirical model with a high correlation factor. A three-point bending test was performed to investigate various multi-axial stress states in actual components.
- A meaningful, intuitive fatigue life prediction model is proposed considering anisotropy as a stress term, which directly utilizes experimental results with a theoretical approach. It can be concluded that the fatigue life of materials with high temperature and anisotropy fiber orientation and polymers can be predicted with reasonable accuracy.
- SEM photography revealed that the higher the temperature and fatigue fracture cycle, the greater the deformation of the polymer matrix, and inversely, the more the deformation of the fiber. The higher the temperature, the more evenly the fiber’s fracture cross-section is.
- The developed numerical model and structural equation are highly consistent between experiments and FEA results. Furthermore, they could accurately export stress and strain as inputs to a semi-empirical model.
- The usefulness of the results proposed in this paper can be outlined in two parts. First, the paper summarizes the static and fatigue behavior considering the anisotropy and temperature of short fiber-reinforced plastic materials, which are increasingly utilized exponentially in the industry. Secondly, it provides insight into the availability of the developed semi-empirical model to predict the fatigue life of CF-PA6.
- The use of FRP affected by temperature and fiber orientation is a remaining challenge for research on much colder temperatures and compressive forces below 0 °C. In addition, using compressive force in testing and investigating the mechanical properties of FRP can accurately describe the complex stress states in industries. Therefore, it can be a better solution to predict the fatigue life and composite use of FRP considering low temperature and compression stress states in the future.

## Figures and Tables

**Table 1:** Mean loads of fatigue tests.

Temperature [°C]	Selected Mean Load for Each Specimen Direction [kN]		
	0°	45°	90°
40	2.000	1.250	1.125
60	1.750	1.125	1.000
100	1.500	1.000	0.750

**Table 2:** Summary of fatigue tests on the ASTM-D 638 fatigue test specimens.

Temperature [°C]	Specimen Angle [°]	Max. Load [kN]	Min. Load [kN]	Mean Load [kN]	Amplitude Load [kN]	*N_f_* [Cycles]
40	0	3.20	0.80	2.00	1.20	1512
		3.40	0.60	2.00	1.40	980
		2.80	1.20	2.00	0.80	3400
		1.40	0.60	1.00	0.40	106,263
		2.40	2.20	2.30	0.10	674,785
	45	1.90	0.60	1.25	0.65	7500
		2.10	0.40	1.25	0.85	2420
		1.70	0.80	1.25	0.45	46,700
		0.90	0.40	0.65	0.25	268,410
		1.60	1.40	1.50	0.10	340,485
	90	1.75	0.50	1.12	0.62	1774
		1.95	0.30	1.12	0.82	1670
		1.55	0.70	1.12	0.42	9800
		0.90	0.30	0.60	0.30	76,000
		1.50	1.30	1.40	0.10	361,807
60	0	2.90	0.60	1.75	1.15	1820
		3.25	0.25	1.75	1.50	770
		2.60	0.90	1.75	0.85	8700
		1.30	0.50	0.90	0.40	79,805
		2.10	1.90	2.00	0.10	892,500
	45	1.75	0.50	1.12	0.62	5840
		1.95	0.30	1.12	0.82	642
		1.55	0.70	1.12	0.42	10,400
		0.80	0.30	0.55	0.25	89,600
		1.50	1.30	1.40	0.10	428,500
	90	1.50	0.50	1.00	0.50	11,650
		1.60	0.40	1.00	0.600	3800
		1.30	0.70	1.00	0.30	51,007
		0.70	0.30	0.50	0.20	346,900
		1.40	1.20	1.30	0.10	276,000
100	0	2.25	0.75	1.50	0.75	4753
		2.50	0.50	1.50	1.00	1670
		2.00	1.00	1.50	0.50	23,060
		1.00	0.40	0.70	0.30	660,650
		1.80	1.60	1.70	0.10	475,439
	45	1.50	0.50	1.00	0.50	4460
		1.60	0.40	1.00	0.60	1790
		1.30	0.70	1.00	0.30	29,800
		0.70	0.30	0.50	0.20	108,699
		1.40	1.20	1.30	0.10	90,335
	90	1.15	0.35	0.75	0.40	5700
		1.35	0.15	0.75	0.60	1213
		1.00	0.50	0.75	0.25	39,355
		0.50	0.20	0.35	0.15	270,883
		1.10	0.90	1.00	0.10	166,068

**Table 3:** Summary of fatigue experiments on the three-point bending specimens.

Temperature [°C]	Specimen Angle [°]	Max. Load [kN]	Min. Load [kN]	Mean Load [kN]	Amplitude Load [kN]	*N_f_* [Cycles]
40	0	0.13	0.11	0.12	0.01	2109
	45	0.10	0.80	0.90	0.01	905
	90	0.08	0.06	0.07	0.01	2160
60	0	0.11	0.09	0.10	0.01	15,041
	45	0.08	0.06	0.07	0.01	9800
	90	0.08	0.06	0.07	0.01	5061
100	0	0.09	0.07	0.08	0.01	1200
	45	0.06	0.04	0.05	0.01	671
	90	0.05	0.03	0.04	0.01	2780

**Table 4:** Parameter and constant definitions for the Ramberg–Osgood model.

Symbol	Definition
*ε_p,eff_*	Effective plastic strain
*T*	Temperature in the environmental chamber in °C
*K*	Strength coefficient
*n*	Hardening exponent
*α_m_*	Weight factor for the fiber direction
*β_m_*	Weight factor for the direction normal to the fibers
*E_m_*	Polymer matrix elastic modulus
*E_f_*	Fiber’s elastic modulus
*λ_m_*_, *I*_	The first eigenvalue of the fiber orientation matrix in the region with strong fiber alignment with the polymer flow

**Table 5:** Ramberg–Osgood model constants for each experimental temperature in injection molding process analysis by AME.

*T* [°C]	*σ*_0_ [MPa]	*n*	*α_m_*	*β_m_*	*E_m_* [GPa]	*E*_f_ [GPa]	*λ_m_*_,*I*_
40	250.10	3.34	18.85	11.33	0.78	60.02	0.85
60	215.76	4.14	7.13	8.40	0.56	30.37	0.85
100	227.12	4.46	16.71	17.65	0.40	30.48	0.85

**Table 6:** Tensile strengths of CF-PA6 in the uniaxial tensile tests.

Temperature [°C]	Tensile Strength of Each Specimen Direction [MPa]		
	0°	45°	90°
40	130.3	101.3	77.2
60	122.6	79.6	60.8
100	98.8	62.8	51.2

## Data Availability

Data are contained within the article.
